# Seasonal Hunger: A Neglected Problem with Proven Solutions

**DOI:** 10.1371/journal.pmed.1000101

**Published:** 2009-06-30

**Authors:** Bapu Vaitla, Stephen Devereux, Samuel Hauenstein Swan

**Affiliations:** 1 Fletcher School, Tufts University, Medford, Massachusetts, United States of America; 2 Institute of Development Studies, University of Sussex, Brighton, United Kingdom; 3 Action Against Hunger–UK, London, United Kingdom

**Summary Points**

- Most of the world's acute hunger and undernutrition occurs not in conflicts and natural disasters but in the annual “hunger season,” the time of year when the previous year’s harvest stocks have dwindled, food prices are high, and jobs are scarce.
- We know what works in fighting seasonal hunger and undernutrition: there are identifiable policy and program successes in contexts around the world, but they often operate on a small scale and in isolation.
- Community-based interventions to treat acute undernutrition and promote growth of preschool children are examples of successful interventions that should be scaled up.
- Global scale-up of a basic “minimum essential” intervention package against seasonal hunger would cost around 0.1% of global GDP and save millions of lives, while protecting millions more from severe illness.
- Focusing on seasonal hunger would be an effective way to leverage resources for the attainment of the hunger-related Millennium Development Goal.

Most of the world's acute hunger and undernutrition occurs not in conflicts and natural disasters but in the annual “hunger season,” the time of year when the previous year’s harvest stocks have dwindled, food prices are high, and jobs are scarce (See Text S1, Note A). Nearly seven out of every ten hungry people in the world, or about six hundred million, are either landless rural laborers or members of small farm households [1]. Many of these six hundred million people live in areas where water or temperature constraints allow only one crop harvest per year. Their poverty is driven by seasonal cycles, worsening especially in the preharvest months (see Text S1, note B) [2]. During this “hunger season” period, household food stocks from the last harvest begin to run out: low production levels, inadequate storage facilities, and accumulated debt all combine to force families to sell or consume their agricultural production well before the new harvest. In many poor rural communities, the majority of families are affected, so mutual support networks are undermined. Household-level food deficits translate to general shortages at the local economy level, so food prices on the open market increase considerably during the hunger season.

At the same time, labor markets are flooded with hungry families seeking work. Even for those lucky enough to find employment, wages are low; and working for wages to obtain food for immediate needs comes at the expense of neglecting one’s own farm, thereby compromising future harvests. The net result is that households are forced to reduce the diversity and quantity of food they consume, setting the stage for macro- and micronutrient deficiencies. In addition, the preharvest hunger season is also often the rainy season, when malaria, diarrheal diseases, and other illnesses strike hard. Sick people—who often require increased energy intake—lose their appetites and, in the face of diarrheal and other diseases, struggle to retain what they have eaten. Illness also reduces the labor productivity of households and imposes additional time and money costs in caring for the sick. The psychological effects of these struggles are also profound, as family members often experience elevated levels of anxiety and stress in the hunger season. Women are especially affected, as their responsibilities often include both income-generating activities and care for infants and children. In sum, the convergence of all these challenges at specific times of year generates seasonal patterns of hunger and undernutrition [3],[4] (see Figure 1 for an illustration from Niger).

**Figure 1:**
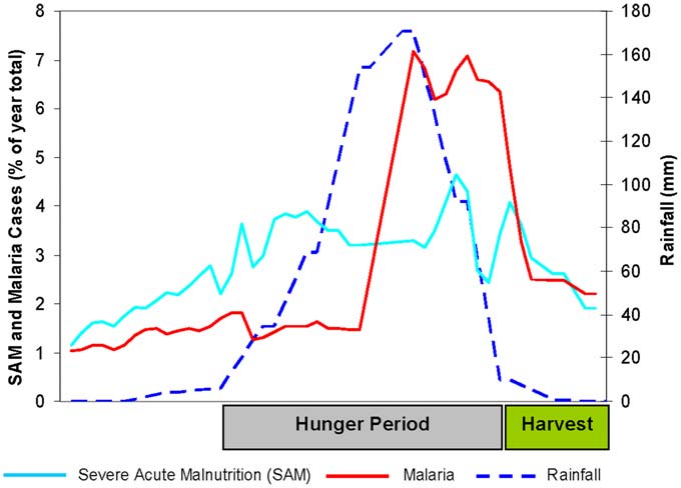
Seasonality in undernutrition, malaria, and rainfall in Niger, 2007 [13]–[15].

## A World Full of Good Ideas

**Figure 2:**
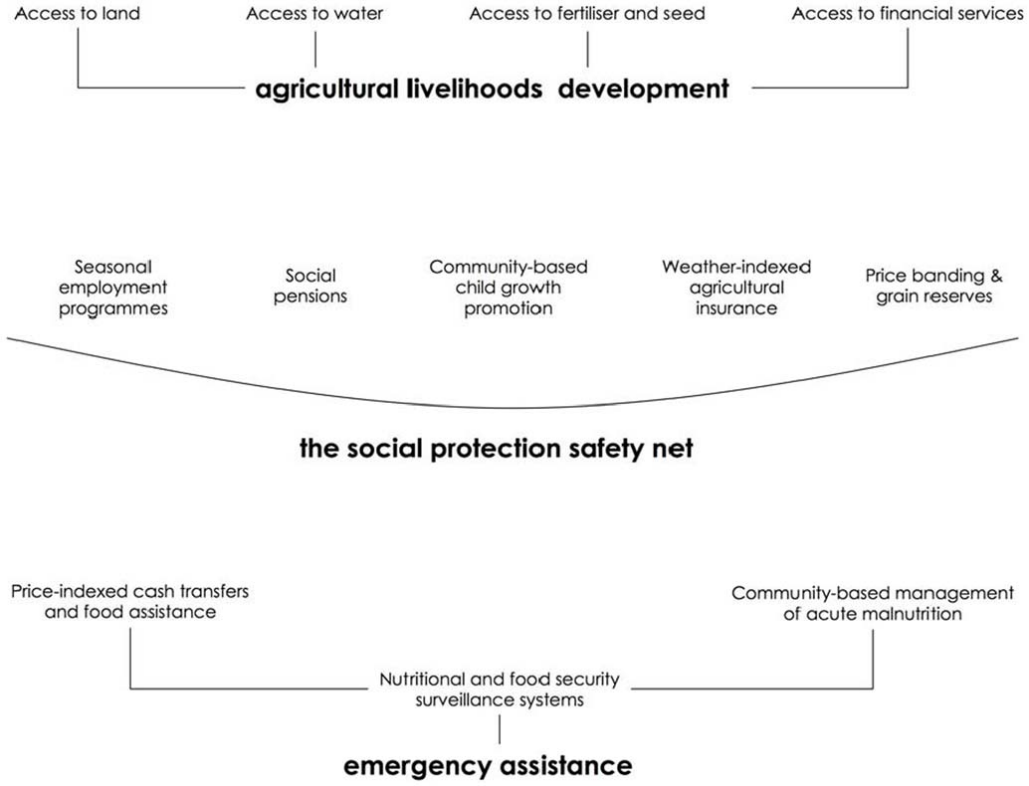
Intervention framework for fighting seasonal hunger.

However, this essay—which summarizes more extended arguments made by the authors in the book *Seasons of Hunger: Fighting Cycles of Quiet Starvation Among the World’s Rural Poor*—argues that seasonal hunger is not inevitable. Governments across the world have experimented with a wide array of policies that have proven successful in combating seasonal hunger. Figure 2 illustrates a selection of these interventions—some of which have been practiced for decades, others being more recent innovations—arranged into categories of “emergency assistance,” “the social protection safety net,” and “rural livelihoods development” [5].

Emergency assistance measures are targeted at people who are suffering from severe or acute hunger and need immediate help. The social protection safety net attempts to prevent vulnerable families from falling into hunger, through a mix of employment, nutrition, food price regulation, and other policies. Finally, rural livelihoods development interventions focus on improving productivity through better access to productive inputs, thereby working towards a future where rural households have incomes high enough—and stable enough—that the social protection safety net will rarely need to be accessed. Taken together, the ideas shown in the diagram represent a highest-priority intervention framework for tackling seasonal hunger. We specifically discuss two initiatives that are particularly relevant to the fields of child nutrition and public health: community-based management of acute malnutrition (CMAM) and child growth promotion (see Text S1, note C).

The CMAM approach is revolutionizing the treatment of severe acute malnutrition among preschool children. Most severe acute malnutrition, a condition which can kill within weeks if not treated, occurs not in “emergency” situations of conflict and natural disaster—as is commonly believed—but rather in contexts of extreme poverty, and particularly during the annual hunger season in rural areas [6]. Traditionally, children suffering from severe acute malnutrition have been treated in hospital-type inpatient settings. However, very high per-patient costs and staffing needs under this approach, as well as the lack of appropriate facilities in many rural areas, mean that only a limited number of undernourished children are fortunate enough to receive treatment. The CMAM approach addresses these issues by mobilizing communities themselves, with the assistance of frontline health workers, to identify and treat the 80% or so of severely acutely undernourished children who do not have other illnesses or complications [7]. The use of easy-to-administer therapeutic foods such as nutrient-dense Ready-to-Use Foods (RUFs) makes this community-based strategy viable. Meanwhile, inpatient care in Therapeutic Feeding Centers (TFCs) can concentrate on the remaining 20% of undernourished children who do have complications.

Although CMAM approaches have been tested on a large scale for only a few years, early results have been impressive. In a survey of 21 CMAM programs in Malawi, Ethiopia, and Sudan between 2001 and 2005, coverage (the percentage of the total under-age-five population screened and treated for undernutrition) increased almost 5-fold over traditional treatment approaches; overall, nearly three-fourths of all children in the project areas were included in the screening. Four out of five children who were treated through CMAM recovered, a rate that compares favorably to inpatient care [8]–[10]. Challenges certainly remain; primary among these the danger that CMAM becomes equated solely with the distribution of therapeutic foods, instead of being understood as a holistic treatment paradigm that requires close supervision by health and nutrition professionals. But there is reason to be optimistic that careful implementation of CMAM will lead to steady improvement in coverage and recovery rates, particularly as screening and treatment schedules become better attuned to the predictable annual cycles of hunger and undernutrition experienced by rural communities across the world.

Child growth promotion initiatives protect preschool children and pregnant/lactating mothers from hunger by integrating a wide variety of health and nutrition services at the village level. The overall objective of growth promotion initiatives is to ensure optimal health and nutrition during the most important child growth periods: during pregnancy and in the first years of life. Seasonal patterns of food deficit or disease that interrupt these critical periods can have permanently adverse developmental consequences. The services commonly found in growth promotion programs include: antenatal care; breastfeeding promotion; health, hygiene, and nutrition education; immunization; child growth monitoring; and supplementary feeding of pregnant women, lactating mothers, and preschool children. While there is currently a spirited debate around the empirical evidence of impact of these interventions, a recent meta-survey of 15 community growth promotion and similar child health/nutrition programs worldwide concludes that, given the presence of certain contextual and program success factors, undernutrition is considerably reduced among enrolled children (see Text S1, note D) [11],[12]. These interventions also have the important additional value of focusing resources on the needs of women; hunger, in its seasonal and other forms, often has a strongly gendered impact, and policies and programs must be careful to do more than just nominally address this issue.

The success of CMAM approaches and child growth promotion initiatives depends on a substantial increase in health care sector investment, particularly in increasing the size and capacity of the community health worker force. Increased investment in these interventions will reap great returns: they are excellent ideas that have proven their potential to save hundreds of thousands of lives and prevent millions of children from becoming ill during the annual hunger season. They deserve to be better funded and more broadly implemented.

## Money and Political Will

Despite the fact that all of the components of the intervention framework above have proven their effectiveness in settings around the world, they are rarely implemented on a large scale or in an integrated manner. This is primarily because “non-emergency” chronic and seasonal hunger have historically occupied a low place on the list of global political priorities, and as a result antihunger efforts do not receive the resources they need to be universally and effectively implemented.

A universal “minimum essential package” against seasonal hunger might emphasize four of the ideas in the intervention framework: CMAM systems, child growth promotion initiatives, seasonal employment programs, and social pensions. Taken together, these four ideas would cover needs among the youngest children, pregnant and lactating mothers, unemployed and underemployed households, and those unable to work. Universal implementation of these four interventions against seasonal hunger would cost in the range of £26–49 billion annually. In a global political environment dominated by fears of severe and protracted recession, prospects for generating additional development assistance on this scale might seem bleak. Yet, put into perspective, this figure amounts to only 0.1% of global GDP, equivalent to four pence a day for each UK resident or 10 cents a day for each US resident. For this small fraction of income, most of the 36 million people who die annually from hunger-related causes—including four million preschool-age children—would be saved.

The political and technical challenges to implementing a broad-based action plan to fight seasonal hunger are admittedly formidable. A good start on the part of governments, donor agencies, and civil society would be to evaluate existing interventions through the analytical lens of seasonality; deploying resources in response to seasonal fluctuations in needs would increase the impact of current programs at little or no additional cost. Such initial “no-cost” success might also open the door for scaling up other proven-effective interventions and eventually generating the resources required to eradicate severe hunger and undernutrition in vulnerable communities across the world.

It is also worth noting that seeking additional assistance to fight seasonal hunger in the present political environment may not be as difficult as it first appears. As noted above, the global food price crisis has brought the issue of hunger back into public view. The Millennium Development Goals deadline of 2015 is approaching and progress towards achieving the hunger-related Goal has been slow; focusing on seasonal hunger could leverage existing funds to accelerate the rate of global hunger reduction. Finally, the world financial crisis is illustrating in dramatic fashion the interdependence of the global economy, and by doing so may be opening the door for a new era of cross-border cooperation. The problems of emerging economies are the problems of the industrialized world, and vice versa; so the implementation of universal social protection safety nets appears more critical than ever. The response of governments worldwide to the financial crisis has proven that political will, and attendant large fiscal commitments, can be generated quickly, if bold leadership is present. The crisis of 25,000 children dying every day from preventable disease and hunger deserves that same sense of urgency.

## Supporting Information

Text S1 Notes for *Seasonal Hunger: A Neglected Problem with Proven Solutions.*
(0.04 MB DOC)

## Abbreviations

CMAM: community-based management of acute malnutrition

## References

[1] United Nations Millennium Project (2005). Halving hunger: It can be done. UN Task Force on Hunger.. http://www.unmillenniumproject.org/reports/tf_hunger.htm.

[2] Ferro-Luzzi A, Branca F, Ulijaszek S, Strickland S (1993). Nutritional seasonality: the dimensions of the problem.. Seasonality and human ecology.

[3] Chambers R, Longhurst R, Pacey A (1981). Seasonal dimensions to rural poverty.

[4] Sahn DE, International Food Policy Research Institute (1989). Seasonal variability in Third World agriculture: The consequences for food security.

[5] Devereux S, Vaitla B, Hauenstein-Swan S (2008). Seasons of hunger: Fighting cycles of quiet starvation among the world's rural poor.

[6] Gross R, Webb P (2006). Wasting time for wasted children: severe child undernutrition must be resolved in non-emergency settings.. Lancet.

[7] World Health OrganizationWorld Food ProgramUnited Nations System Standing Committee on NutritionUnited Nations Children’s Fund (2007). Community-Based Management of severe acute malnutrition.. http://www.who.int/nutrition/topics/severemalnutrition_statement_commbased/en/index.html.

[8] Collins S, Sadler K, Dent N, Khara T, Guerrero S (2005). Key issues in the success of community-based management of severe malnutrition.. Food Nutr Bull.

[9] Collins S, Dent N, Binns P, Bahwere P, Sadler K (2006). Management of severe acute malnutrition in children.. Lancet.

[10] Sadler K (2006). Treating acute malnutrition seriously.. http://www.nutrition.tufts.edu/docs/media/Sadler_10_10_07.ppt.

[11] Hunt J (2005). The potential impact of reducing global malnutrition on poverty reduction and economic development.. Asia Pac J Clin Nutr.

[12] Mason J, Soekirman-Galloway R, Martines J, Musgrove P, Sanders D, Jamison D, Breman JG, Measham AR, Alleyne G, Claeson M (2006). Community Health and Nutrition Programmes.. Disease control priorities in developing countries.

[13] FEWS NET (2008). Niger. Seasonal Calendar and Critical Events Timeline.. http://www.fews.net/PAGES/TIMELINEVIEW.ASPX?GB=NE&TLN=EN&L=EN.

[14] WHO (2007). Bulletins hebdomadaire de morbidité, de mortalité et de surveillance nutritionnelle au Niger (week 1–week 52, 2007).. http://www.who.int/hac/crises/ner/sitreps/rapport_hebdomadaire/en/index.html.

[15] WMO (2008). Mean total precipitation for the period 1961–1990.. http://www.worldweather.org/074/c00327.htm.

