# Supplementary material for: Seasonal Hunger: A Neglected Problem with Proven Solutions
Source: PLoS Med. 2009 Jun 30;6(6):e1000101. doi: 10.1371/journal.pmed.1000101 (PMC2696035; doi:10.1371/journal.pmed.1000101)
Supplement: Text S1 — Notes for Seasonal Hunger: A Neglected Problem with Proven Solutions. (0.04 MB DOC) [file pmed.1000101.s001.doc]

Supporting text S1

A. The terms “hunger” and “undernutrition” are employed with varying meanings in the academic and popular literature. Throughout this article, we use “hunger” to mean a general shortage of food at the household level (as compared to needs) and “undernutrition” to signify the physiological condition of nutritional deficiency. The latter is usually - and in this paper - used in reference to children assessed by anthropometric measurement.

B. Lack of reliable agro-climatic, food consumption and nutritional data make exact estimates of the number affected by seasonal hunger difficult. Ferro-Luzzi and Branca (1987) estimate a global figure of at least 300 million people who "should be considered at risk for functional and metabolic impairment following their exposure to seasonal bottlenecks in energy turnover that exceed the body's physiological tolerance" (p.162). However, their analysis is limited to: a) adults only; b) primary undernutrition caused by insufficient food intake, and not secondary undernutrition caused by seasonal disease which limits nutrient absorption and retention; and c) a limited set of countries – 19 in Sub-Saharan Africa, plus India and China. Expanding the analysis to cover children, undernutrition caused by disease, and a larger set of countries would likely expand the figure of 300 million considerably. In any case, those affected by seasonal hunger comprise the majority, probably the vast majority, of the 600 million rural people in the world who are estimated to be hungry. In addition, the remainder – those who suffer from year-round 'chronic' hunger – are not immune to seasonal cycles. Insofar as they are dependent on the agricultural economy, their hunger also deepens in the pre-harvest months.

C. Strictly speaking, CMAM interventions are focused on undernutrition, not the more general condition of “malnutrition”, which includes all forms of nutritional disorders, including obesity. However, following convention with regard to these interventions, we use the “CMAM” acronym and the phrase “severe acute malnutrition” in the following section, but our focus remains solely on undernutrition.

D. Most important among the success factors are: commitment to nutrition at all political levels; participatory planning with the community; the involvement of charismatic community leaders; strong investment in management; health/nutrition awareness training; setting time-bound objectives; and the involvement of local NGOs. See Hunt (2005) and Mason et al. (2006) for a detailed discussion about the potentials and constraints to effectiveness of various child growth promotion interventions.
